# Role of Survival Post-Progression in Phase III Trials of Systemic Chemotherapy in Advanced Non-Small-Cell Lung Cancer: A Systematic Review

**DOI:** 10.1371/journal.pone.0026646

**Published:** 2011-11-17

**Authors:** Katsuyuki Hotta, Katsuyuki Kiura, Yoshiro Fujiwara, Nagio Takigawa, Akiko Hisamoto, Eiki Ichihara, Masahiro Tabata, Mitsune Tanimoto

**Affiliations:** Department of Respiratory Medicine, Okayama University Hospital, Okayama, Japan; Queen Elizabeth Hospital, Hong Kong

## Abstract

**Background:**

In advanced non-small-cell lung cancer (NSCLC), with the increasing number of active compounds available in salvage settings, survival after progression to first-line chemotherapy seems to have improved. A literature survey was conducted to examine whether survival post-progression (SPP) has improved over the years and to what degree SPP correlates with overall survival (OS).

**Methods and Findings:**

Median progression-free survival (MPFS) time and median survival time (MST) were extracted in phase III trials of first-line chemotherapy for advanced NSCLC. SPP was pragmatically defined as the time interval of MST minus MPFS. The relationship between MPFS and MST was modeled in a linear function. We used the coefficient of determination (*r* ^2^) to assess the correlation between them. Seventy trials with 145 chemotherapy arms were identified. Overall, median SPP was 4.7 months, and a steady improvement in SPP was observed over the 20 years (9.414-day increase per year; p<0.001) in parallel to the increase in MST (11.253-day increase per year; p<0.001); MPFS improved little (1.863-day increase per year). Overall, a stronger association was observed between MST and SPP (*r* ^2^ = 0.8917) than MST and MPFS time (*r* ^2^ = 0.2563), suggesting SPP and MPFS could account for 89% and 25% of the variation in MST, respectively. The association between MST and SPP became closer over the years (*r* ^2^ = 0.4428, 0.7242, and 0.9081 in 1988–1994, 1995–2001, and 2002–2007, respectively).

**Conclusions:**

SPP has become more closely associated with OS, potentially because of intensive post-study treatments. Even in advanced NSCLC, a PFS advantage is unlikely to be associated with an OS advantage any longer due to this increasing impact of SPP on OS, and that the prolongation of SPP might limit the original role of OS for assessing true efficacy derived from early-line chemotherapy in future clinical trials.

## Introduction

Non-small-cell lung cancer (NSCLC) accounts for approximately 75% of all lung cancer cases [1]. The majority of patients with NSCLC have inoperable locally advanced or metastatic disease at the time of diagnosis. The standard treatment for advanced NSCLC has been platinum-based chemotherapy [2]–[4], which, unfortunately, produces a median survival time (MST) of only approximately 1 year [5]–[7]. In contrast, during the last decade, several effective chemotherapeutic agents have been developed for advanced NSCLC and have been shown to yield significant survival advantages ,even in salvage settings [8]–[13].

Given its objectivity and the benefits derived by patients, overall survival (OS) has been historically considered the most important therapeutic objective in advanced NSCLC, whereas progression-free survival (PFS) captures tumor shrinkage, tumor stabilization, and their duration, all of which are essential for evaluation of new target agents [14]. Currently, however, with the increasing number of the aforementioned factors, the effects of subsequent therapies may have the potential to affect the PFS advantage of early-line therapies on OS advantage.

To date, few studies have addressed whether survival after progression to first-line chemotherapy (survival post-progression [SPP]) has substantially improved over the years and to what degree SPP correlates with OS. SPP was first reported in 2009 [15] with use of a simple device. That is, OS was partitioned into two parts by expressing it as the sum of PFS and this “survival postprogression” (SPP) [ie, OS = PFS+(OS−PFS)] [15]. Here, the standard definition of “progression” included death from any cause and so the progression event may be death.

Based on the backgrounds, we conducted a literature survey to address these clinical questions using an abstracted database of randomized phase III trials of systemic first-line chemotherapy for advanced NSCLC.

## Methods

### Eligibility criteria, information sources and search for trials

A literature search was conducted for trials reported between January 1991 and November 2010. To avoid publication bias, both published and unpublished trials were identified through a computer-based search of both the PubMed database and abstracts from the past 10 conferences of the American Society of Clinical Oncology, European Society for Medical Oncology, and International Association of Study on Lung Cancer. The following search terms were used: “lung neoplasm,” “carcinoma,” “non-small cell,” “chemotherapy,” and “randomized controlled trial.” The search was also guided by a thorough examination of reference lists from original and review articles, relevant books, meeting abstracts, and the Physician Data Query registry of clinical trials.

### Study selection

Phase III trials were eligible if they evaluated first-line systemic chemotherapy for advanced or metastatic NSCLC. Among chemotherapeutic agents, new agents were defined previously as those including docetaxel, paclitaxel, vinorelbine, gemcitabine, and irinotecan, while old agents were defined as those that had been developed before these new agents were introduced clinically (etoposide, ifosfamide, vindesine, vinblastine) [16]. Drugs thought to act on known specific molecular targets, such as tyrosine kinase inhibitors (TKIs), neutralizing antibodies, anti-angiogenic agents, matrix metalloproteinase inhibitors, and antisense oligonucleotides, were defined as molecular-targeted agents [17]. Trials that provided data for median PFS (MPFS) and MST in each report were included. Trials that were designed to assess combined modality treatments, including radiotherapy and surgery, were excluded. Clinical trials of salvage chemotherapy (second-line or later setting) were also ineligible.

### Data collection process and data items

To avoid bias in the data abstraction process, two medical oncologists (Y.F. and K.H.), one of whom (K.H.) is board-certified in medical oncology, independently abstracted the data from the trials and subsequently compared their results, as described previously [18]–[25]. The following information was obtained from each report: year of trial initiation, number of patients randomized, treatment regimens, publication type, and primary endpoint. MPFS and MST were also extracted from each report. Here, SPP was defined as the MST minus the MPFS for each trial arm, based on previous reports [26], [27].

All data were checked for internal consistency. Disagreements were resolved by discussions among the investigators, although their frequencies and patterns were not formally recorded. Principal investigators of the trials were contacted to confirm or update the published data.

### Summary measures and synthesis of results

Data from the phase III trials were evaluated through linear regression analysis, by assigning a weight equal to the sample size to each trial. The strength of associations was defined *a priori* using the commonly accepted criteria for the coefficient of determination (*r* ^2^); briefly, it gives the proportion of the variance of one variable that is predictable from the other variable. It is a measure that allows for the determination of how certain one can be in making predictions from a certain model. The coefficient of determination is such that 0≤*r* ^2^≤1, and a higher *r* ^2^ score indicates a stronger association. Correlations were described graphically through bubble plots in which each bubble represents a pair of arms with size proportional to the sample size of each trial. To examine possible differential associations between MST and MPFS and between MST and SPP, the analysis was repeatedly conducted after stratifying several clinical factors (Table 4). Differential associations were then evaluated by entering multiplicative interaction terms between each factor.

All p values were from two-sided tests, and significance was set at p<0.05. Statistical analyses were conducted using the STATA software (ver. 10; StataCorp, College Station, TX, USA).

## Results

### Trial demographics

**Figure 1:**
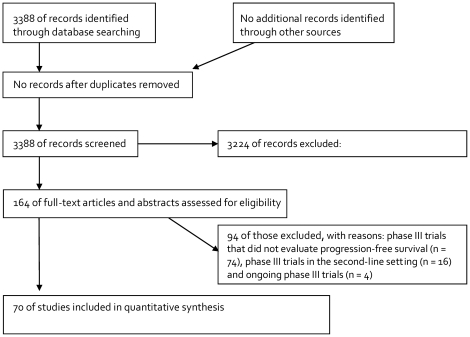
The PRISMA flow chart showing the progress of trials through the review.

Of the 3388 trials screened, 70 phase III trials (Table 1 and File S1) initiated between 1988 and 2007 involving 38,721 patients with advanced NSCLC were identified as having data regarding survival data (Fig. 1). Sixty-four, five, and one of the 70 trials had two, three, and four treatment arms, respectively, while we excluded two best supportive care only arms. Finally, in total, 145 chemotherapeutic treatment arms with 34,501 randomly allocated patients were eligible for this study. Trial characteristics and chemotherapeutic regimens investigated are listed in Tables 1 and 2, respectively.

**Table 1:** Trial demographics in 70 trials.

No. of randomly assigned patients per trial (median; range)	403 (126–1725)
Year of trial initiation (median; range)	2002 (1988–2007)
Publication type (full text/abstract form only)	55/15
Primary endpoint (OS/PFS)	47/23
Proportion of pts with ECOG PS of 0 and 1 (median; range)	96 (0–100)*

Abbreviations: OS  =  overall survival, PFS  =  progression-free survival, ECOG PS  =  Eastern Cooperative Oncology Group performance status.

*indicating the median score of proportion of good ECOG-PS patients in each eligible trial and its range.

**Table 2:** Characteristics of 145 chemotherapy arms in the 70 trials.

	No. of treatment arms
Platinum-based regimens	110* (75.9%)
− cisplatin-based	63 (43.4%)
+ new agent‡	33 (22.8%)
+ new agent + molecular-targeted agent‡ #	13 (9.0%)
+ other	17 (11.7%)
− carboplatin-based	53 (36.6%)
+ new agent‡	39 (26.9%)
+ new agent + molecular-targeted agent‡ #	12 (8.3%)
+ other	2 (1.4%)
− oxaliplatin-based	1 (0.7%)
Non-platinum regimens	35 (24.1%)
− monotherapy	18 (12.4%)
− combination therapy	17 (11.7%)

*In seven arms, cisplatin or carboplatin was investigated.

‡ New agent was defined as those including docetaxel, paclitaxel, vinorelbine, gemcitabine, and irinotecan (see Methods section).

# Molecular-targeted agent was defined as agents acting on known specific molecular targets, such as tyrosine kinase inhibitors, neutralizing antibodies, and antisense oligonucleotides (see Methods section).

### Trend in survival times of patients enrolled into phase III trials

**Figure 2:**
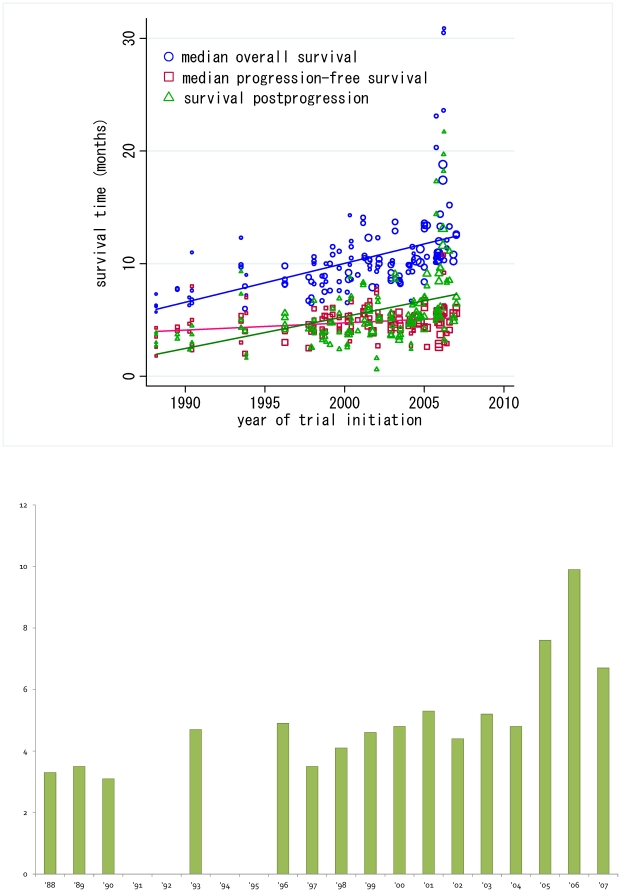
Time trends in survival data. A. Trend in survival times in advanced NSCLC patients enrolled into phase III trials. Median survival time (0.3751-month [11.253-day] increase per year; p<0.001; blue), median progression-free survival time (0.0621-month [1.863-day] increase per year; p = 0.006; pink), survival post-progression (0.3138-month [9.414-day] increase per year; p<0.001; green). All analyses were weighted by trial size. Y-axis indicates survival time of each endpoint (months). B. Absolute mean SPP value per year. X-axis and Y-axis indicate year of trial initiation and mean SPP value (months) in each year, respectively.

This study focused on the trend in survival time of patients during the study period. Median SPP in the whole arm was 4.7 months. As seen in Fig. 2A and 2B, a scattergram demonstrates the progressive improvement in the MST of advanced NSCLC patients enrolled into phase III trials over the years with a 0.3751-month (11.253-day) increase per year (p<0.0001; blue). Indeed, SPP was prolonged with 9 months in more recent trials that were initiated in 2006 or 2007. Additionally, slopes of the fitted lines of SPP (0.3138-month [9.414-day] increase per year, p<0.0001; green) and MST (blue) were nearly parallel despite a small improvement in MPFS (0.0621-month [1.863-day] increase per year; pink), indicating that the gain in MST may be primarily attributable to the increase in SPP rather than in MPFS.

### Factors affecting SPP

Next, a multiple regression analysis for SPP was conducted to clarify which clinical factors could affect SPP (Table 3). The year of trial initiation was a significant factor (regression coefficient of 0.2776; p<0.001), indicating that SPP has steadily increased over the years even after adjusting other covariates listed in Table 3. Additionally, a longer SPP time was associated with several clinical situations, including a high proportion of good PS patients (p = 0.002) and first-line use of monotherapy (p = 0.011) and molecular-targeted agent (p = 0.025).

**Table 3:** Multiple regression analysis for survival post-progression (SPP).

Covariates	Regression coefficient	p-value
Year of trial initiation	0.2776	<0.001
Proportion of patients with performance status of 0 to 1	0.0536	0.002
Platinum use (yes vs. no)	−0.6326	0.299
No. of chemotherapeutic agents combined		
(single vs. doublet)	2.1705	0.011
(triplet or quartet vs. doublet)	−0.6014	0.200
Use of older agents (yes vs. no)¶	1.7061	0.070
Use of molecular-targeted agents (yes vs. no)§	1.0555	0.025

¶ Older agents were defined as those that had been developed before newer drugs (i.e., docetaxel, paclitaxel, vinorelbine, gemcitabine, irinotecan) were introduced clinically (see Methods section).

§ Defined as agents acting on known specific molecular targets, such as tyrosine kinase inhibitors, neutralizing antibodies, matrix metalloproteinase inhibitors, and antisense oligonucleotides (see Methods section).

### Associations between MST and MPFS and between MST and SPP

**Figure 3:**
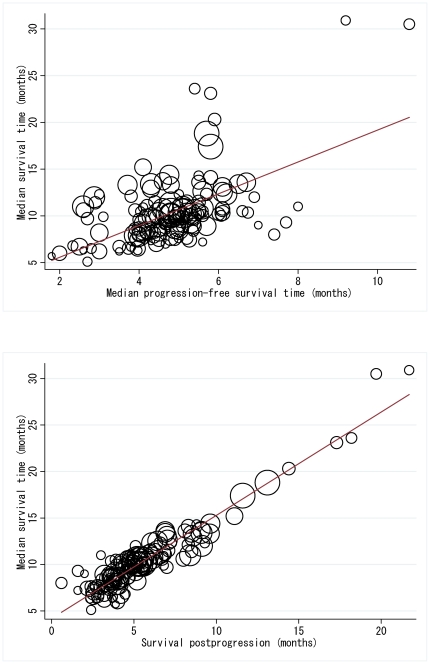
Overall survival, progression-free survival and survival post-progression. A. Associations between median survival time and median progression-free survival time (*r* ^2^ = 0.2563). B. Associations between median survival time and survival post-progression (*r* ^2^ = 0.8917). All analyses were weighted by trial size.

MST and MPFS, and MST and SPP were plotted among the 145 chemotherapeutic arms. Overall, MST and MPFS were weakly associated (*r* ^2^ = 0.2563), suggesting that MPFS explained only 25.6% of the overall variability in MST (Fig. 3A). Interestingly, however, the regression analysis revealed that several clinical situations strengthened the association, such as when first-line, platinum-based chemotherapy was investigated (*r* ^2^ = 0.7354) compared with the situation in which agents other than platinum were investigated (*r* ^2^ = 0.0849; p for interaction <0.001; Table 4). In contrast, SPP was strongly associated with MST (*r* ^2^ = 0.8917), meaning that it could account for as much as 89% of the variation in MST (Fig. 3B).

**Table 4:** Associations between median survival time and median progression-free survival or survival post-progression.

	No. of treatment arms	MST vs. MPFS		MST vs. SPP	
Factors		*r* ^2^	p (interaction)*	*r* ^2^	p (interaction)*
**<Type of anti-cancer agent >**					
Platinum-based chemotherapy					
yes	109	0.0849		0.8451	
no	35	0.7354	<0.001	0.9699	<0.001
Use of old chemotherapeutic agents					
yes	24	0.2095		0.4986	
no	120	0.2706	0.172	0.8970	0.123
Use of molecular-targeted agents					
yes	42	0.3012		0.9208	
no	102	0.2075	0.045	0.8058	0.196
**<Trial design/characteristics>**					
Primary endpoint (overall survival)					
yes	95	0.2156		0.8652	
no	43	0.3842	0.137	0.9038	0.175
Publication type (full text)					
yes	115	0.2996		0.8956	
no	29	0.1199	0.238	0.8827	0.211
Year of trial initiation§					
1988∼1994	20	0.3266		0.4428	
1995∼2001	51	0.4214	0.517	0.7242	0.215
2002∼2007	71	0.2319	0.220	0.9081	0.227

Abbreviations: MST  =  median survival time, MPFS  =  median progression-free survival, SPP  =  survival post-progression.

§ The trials, initiated between 1988 and 2007, were simply divided into 3 generations on the basis of the year of trial initiation.

*We entered multiplicative interaction terms between each factor to assess the differential associations compared with each of the first-row categories.

How the year of trial initiation affected the associations between MST and MPFS and between MST and SPP was also examined. The trials, initiated between 1988 and 2007, were simply divided into three generations on the basis of the year of trial initiation. Each period was considered as follows: the early period when old cytotoxic agents and cisplatin were primarily investigated (1988–1994), the mid period when new cytotoxic agents and carboplatin were introduced in phase III trials (1995–2001), and the late period when molecular-targeted agents were introduced in phase III trials (2002–2007). Despite there being no significant p-values for an interaction, the association between MST and MPFS seemed stably weak or a little bit weaker with the passing of the years, while SPP became more strongly correlated with MST with time (Table 4).

## Discussion

This study investigated whether SPP, defined here as MST minus MPFS for each trial arm [26], [27], has improved substantially over the years and to what degree SPP correlates with OS. We showed a steady improvement in SPP over the past 20 years (9.414-day increase per year), in parallel with the increase in MST (11.253-day increase per year), while MPFS improved less (1.863-day increase per year). MST was strongly associated with SPP time (*r* ^2^ = 0.8917), not with MPFS time (*r* ^2^ = 0.2563). The association between MST and SPP became stronger over the observed period (*r* ^2^ = 0.4428, 0.7242, and 0.9081 in 1988–1994, 1995–2001, and 2002–2007, respectively). Longer SPP time was also associated with several clinical situations, including first-line use of molecular-targeted agents.

Because almost all patients with advanced NSCLC will suffer from progression of their disease, the ultimate goal of palliative chemotherapy is prolongation of OS as well as improvement in patients' symptoms and quality of life. Thus, the use of OS to assess the efficacy of chemotherapies for advanced NSCLC seems justified. Recently, however, there has been a growing debate on the use of OS as the primary endpoint in oncological clinical trials [28]. This debate has been going on for several years, especially in cases of colorectal cancer [29]–[30]. In advanced colorectal cancer, OS has been considered an insensitive efficacy criterion because potentially active subsequent therapies are not controlled in most randomized trials; OS may be increased or decreased by such therapies [31]. In this situation, it is naturally supposed that crossover would dilute and skew the true OS difference; thus, no or few observed OS differences would not always indicate a lack of survival advantage of the new compound if it goes beyond certain boundaries [15].

From another point of view, Broglio et al. stressed through their simulation study, the importance of SPP in understanding treatment effects for metastatic cancers [15]. In their study, when the median SPP was small, there was usually a statistically significant benefit in OS when there was a statistically significant treatment benefit in PFS. In contrast, longer periods of SPP added randomness, diluting the treatment effect and making statistical significance in OS decreasingly likely. Looking back on advanced NSCLC, recent observations suggest that the use of effective salvage therapies extends SPP in advanced NSCLC [9]–[13]. Additionally, we indeed found a gain in SPP over the years in the current study (Fig. 2). Thus, the results of Broglios' simulation study could be applied to recent clinical trial settings in advanced NSCLC.

This study had several limitations. All analyses were conducted using abstracted data, but without individual patient data (IPD). Trial-level data, as described here, are not necessarily linked to individual-level data, so our data cannot always be used to predict an individual's chance of survival on the basis of MPFS or SPP shown here. Further IPD analysis will be performed to confirm the current observations [32]. Also, this type of study retrospectively analyzes somewhat heterogeneous data, meaning that study results seem speculative, not definitive. Another critical problem is that the incremental gain in survival (PFS and MST), rather than formal parameters, proportional or absolute risk of events, was applied here because a limited number of trials have reported hazard ratios and thus predictions based on hazard ratio would not be representative and could be biased. SPP was also used, the definition of which has not been fully validated, but has been used in previous reports [25], [26]. These pragmatic approaches seem easy to understand for clinicians involved in NSCLC treatment, but the results obtained here are rather hypothesis-generating, and thus remain to be confirmed by other studies using more formal parameters. Furthermore, information of post-study chemotherapies and supportive care in each trial could not be obtained; thus, details of why SPP time was prolonged remain unknown. Finally, publication bias is a significant threat to the validity of such analysis because it is difficult to completely rule out this possibility. Thus, trials that had not yet been published as well as those that had already been published were collected. All of these issues could have potentially biased the present findings, and the results should be interpreted cautiously.

In conclusion, this study demonstrated that even in advanced NSCLC, SPP, rather than PFS, has became more strongly associated with OS over the years, potentially because of intensive post-study treatments. Due to this increasing impact of SPP on OS, even in advanced NSCLC, a PFS advantage seems hardly associated with an OS advantage any longer. This indicates that the prolongation of SPP might limit the classical role of OS for assessing true efficacy derived from early-line chemotherapy in future clinical trials.

## Supporting Information

File S1 **Trial demographics in 70 trials.** Abbreviations: F = full text, A = abstract form only, pts = patients, PS = performance status, OS = overall survival. Good PS indicates PS of 0 and 1.
(DOC)
