# Supplementary material for: Role of Survival Post-Progression in Phase III Trials of Systemic Chemotherapy in Advanced Non-Small-Cell Lung Cancer: A Systematic Review
Source: PLoS One. 2011 Nov 17;6(11):e26646. doi: 10.1371/journal.pone.0026646 (PMC3219633; doi:10.1371/journal.pone.0026646)
Supplement: File S1 — Trial demographics in 70 trials. Abbreviations: F = full text, A = abstract form only, pts = patients, PS = performance status, OS = overall survival. Good PS indicates PS of 0 and 1. (DOC) [file pone.0026646.s001.doc]

File S1

Webappendix

Trial demographics in 70 trials

| Authors | Type of reporting | Primary endpoint | Published year | Year of study initiation | No. of enrolled pts | No. of randomized pts | Proportion of pts with good PS (%) |
| --- | --- | --- | --- | --- | --- | --- | --- |
| Splinter TA | F | others | 1996 | 1988 | 225 | 211 | 82 |
| Kosty MP | F | OS | 1994 | 1989 | 291 | 266 | 100 |
| Buccheri G | F | OS | 1994 | 1990 | 151 | 151 | 60 |
| Ianniello GP | F | others | 1996 | 1990 | 158 | 158 | 65 |
| Gridelli C | F | others | 1996 | 1990 | 204 | 204 | 73 |
| Giaccone G | F | OS | 1998 | 1993 | 332 | 332 | 85 |
| Font A | F | others | 1999 | 1993 | 126 | 126 | 87 |
| Westeel V | F | OS | 2005 | 1993 | 227 | 181 | 88 |
| Wozniak AJ | F | OS | 1998 | 1993 | 432 | 415 | 100 |
| Rosell R | F | others | 2002 | 1996 | 618 | 618 | 83 |
| Kelly K | F | OS | 2001 | 1996 | 444 | 408 | 100 |
| Gridelli C | F | OS | 2003 | 1997 | 759 | 707 | 80 |
| Lilenbaum RC | F | OS | 2005 | 1997 | 584 | 561 | 82 |
| Souquet PJ | F | OS | 2002 | 1998 | 259 | 259 | 64 |
| Gridelli C | F | others | 2003 | 1998 | 526 | 503 | 87 |
| Smit EF | F | OS | 2003 | 1998 | 480 | 480 | 88 |
| Wachters FM | F | others | 2003 | 1998 | 240 | 240 | 88 |
| Greco FA | F | OS | 2007 | 1998 | 337 | 337 | 92 |
| Laack E | F | OS | 2004 | 1999 | 300 | 300 | 81 |
| Rudd RM | F | OS | 2005 | 1999 | 422 | 422 | 86 |
| Pujol JL | F | others | 2005 | 1999 | 311 | 311 | 92 |
| Bissett D | F | OS | 2005 | 1999 | 362 | 362 | 100 |
| von Plessen C | F | others | 2006 | 2000 | 300 | 300 | 70 |
| Leighl NB | F | OS | 2005 | 2000 | 774 | 774 | 88 |
| M. Ballardini | A | OS | 2009 | 2000 | 241 | 241 | 94 |
| Kudoh S | F | OS | 2006 | 2000 | 182 | 182 | 95 |
| Williamson SK | F | OS | 2005 | 2000 | 397 | 367 | 100 |
| Tan E | F | others | 2005 | 2000 | 316 | 316 | 100 |
| Rigas JR | A | OS | 2004 | 2001 | 930 | 930 | 86 |
| Sandler A | F | OS | 2006 | 2001 | 878 | 878 | 96 |
| Cobo M | F | others | 2007 | 2001 | 444 | 366 | 100 |
| Comella P | F | OS | 2007 | 2001 | 449 | 433 | 100 |
| Kubota K | F | OS | 2006 | 2001 | 401 | 393 | 100 |
| Belani CP | A | OS | 2010 | 2002 | 519 | 255 | 44 |
| Fidias PM | F | OS | 2009 | 2002 | 566 | 309 | 92 |
| Paz-Ares L | F | OS | 2006 | 2002 | 670 | 670 | 100 |
| Blumenschein GR | F | OS | 2005 | 2002 | 612 | 612 | 100 |
| Ramlau R | F | OS | 2005 | 2002 | 623 | 623 | 100 |
| Lee SM | F | OS | 2009 | 2003 | 722 | 722 | 90 |
| H. Groen | A | OS | 2009 | 2003 | 561 | 561 | 94 |
| Takeda K | F | OS | 2009 | 2003 | 604 | 604 | 99 |
| Gridelli C | F | OS | 2007 | 2003 | 400 | 299 | 100 |
| Reynolds C | A | OS | 2007 | 2004 | 202 | 170 | 0 |
| Pirker R | F | OS | 2008 | 2004 | 1688 | 1125 | 83 |
| Gaafar RM | A | OS | 2010 | 2004 | 173 | 173 | 94 |
| Scagliotti GV | F | OS | 2008 | 2004 | 1833 | 1725 | 100 |
| Weissman CH | A | others | 2008 | 2004 | 383 | 383 | 100 |
| Tan EH | F | others | 2009 | 2004 | 390 | 390 | 100 |
| P. A. Kosmidis | F | OS | 2009 | 2004 | 415 | 398 | 100 |
| W. Schuette | A | others | 2009 | 2004 | 192 | 192 | NR |
| Reynolds C | F | OS | 2009 | 2004 | 202 | 170 | NR |
| Manegold C | F | OS | 2010 | 2005 | 839 | 839 | 96 |
| Lynch T | F | others | 2007 | 2005 | 676 | 676 | 98 |
| Ciuleanu T | F | others | 2009 | 2005 | 745 | 663 | 100 |
| Reck M | F | others | 2007 | 2005 | 1050 | 1043 | 100 |
| Hirsh V | A | OS | 2008 | 2005 | 828 | 828 | 100 |
| Cappuzzo F | F | others | 2009 | 2005 | 1949 | 889 | 100 |
| Goss GD | F | OS | 2009 | 2005 | 296 | 251 | 100 |
| Lee JS | A | OS | 2009 | 2005 | 309 | 309 | NR |
| Quoix EA | A | OS | 2010 | 2006 | 451 | 451 | 73 |
| Mok TS | F | others | 2009 | 2006 | 1329 | 1217 | 90 |
| Perol M | A | others | 2010 | 2006 | 834 | 464 | 95 |
| Mitsudomi T | F | others | 2009 | 2006 | 337 | 177 | 97 |
| Maemondo M | F | others | 2009 | 2006 | 230 | 230 | 98 |
| Kabbinavar FF | A | others | 2010 | 2006 | 1160 | 768 | 100 |
| Scagliotti G | F | OS | 2010 | 2006 | 1043 | 926 | 100 |
| Fidias P | A | OS | 2010 | 2006 | 903 | 903 | 100 |
| Okamoto I | F | OS | 2010 | 2006 | 564 | 563 | 100 |
| Gatzemeier | A | OS | 2010 | 2007 | 904 | 772 | 100 |
| O'Brien ME | F | OS | 2004 | NR | 419 | 419 | NR |

Abbreviations: F=full text, A=abstract form only, pts=patients, PS=performance status, OS=overall survival.

Good PS indicates PS of 0 and 1.
